# Rice Yield Estimation Based on Continuous Wavelet Transform With Multiple Growth Periods

**DOI:** 10.3389/fpls.2022.931789

**Published:** 2022-07-01

**Authors:** Chen Gu, Shu Ji, Xiaobo Xi, Zhenghua Zhang, Qingqing Hong, Zhongyang Huo, Wenxi Li, Wei Mao, Haitao Zhao, Ruihong Zhang, Bin Li, Changwei Tan

**Affiliations:** ^1^Jiangsu Key Laboratory of Crop Genetics and Physiology, Jiangsu Co-Innovation Center for Modern Production Technology of Grain Crops, Joint International Research Laboratory of Agriculture and Agri-Product Safety of the Ministry of Education of China, Key Laboratory of Cultivated Land Quality Monitoring and Evaluation (Jiangsu) Ministry of Agriculture and Rural Affairs, Jiangsu Engineering Centre for Modern Agricultural Machinery and Agronomy Technology, Jiangsu Province Engineering Research Center of Knowledge Management and Intelligent Service, Yangzhou University, Yangzhou, China; ^2^Station of Land Protection of Yangzhou City, Yangzhou, China

**Keywords:** remote sensing, hyperspectral, yield, wavelet transform, multi-growth stage, rice

## Abstract

Yield is an important indicator in evaluating rice planting, and it is the collective result of various factors over multiple growth stages. To achieve a large-scale accurate prediction of rice yield, based on yield estimation models using a single growth stage and conventional spectral transformation methods, this study introduced the continuous wavelet transform algorithm and constructed models under the premise of combined multiple growth stages. In this study, canopy reflectance spectra at four important stages of rice elongation, heading, flowering and milky were selected, and then, a rice yield estimation model was constructed by combining vegetation index, first derivative and wavelet transform based on random forest algorithm or multiple stepwise regression. This study found that the combination of multiple growth stages significantly improved the model accuracy. In addition, after two validations, the optimal model combination for rice yield estimation is first derivative-wavelet transform-vegetation index-random forest model based on four growth stages, with the coefficient of determination (R^2^) of 0.86, the root mean square error (RMSE) of 35.50 g·m^−2^ and the mean absolute percentage error (MAPE) of 4.6% for the training set, R^2^ of 0.85, RMSE of 33.40 g.m^−2^ and MAPE 4.30% for the validation set 1, and R^2^ of 0.80, RMSE of 37.40 g·m^−2^ and MAPE of 4.60% for the validation set 2. The research results demonstrated that the established model could accurately predict rice yield, providing technical support and a foundation for large-scale statistical estimating of rice yield.

## Introduction

Rice is one of the important food crops in China and occupies an important position in agricultural production, so the production work of rice is also related to our food security and sustainable agricultural development. In recent years, with the improvement of people’s economic level, people’s research on rice has gradually shifted to the quality aspect, but the yield is still an aspect that we cannot ignore. Large-scale estimating of rice yield is of great importance to ensure national food security and regulate food crop production.

The information of different bands in spectral data, the vegetation indices, and hyperspectral characteristic parameters of various band combinations can directly or indirectly tell the growth status of crops, and they are a comprehensive reflection of the effects of various factors on field crops (Curran, 1989). The research of hyperspectral technology on rice focuses on using various independent variables such as original reflectance spectrum, differential transformation, vegetation index, area variable, and location to initially establish prediction models for rice leaf area index (LAI), biomass, yield, etc., and then achieves rice yield estimates (Feng et al., 2021; Miclea et al., 2022). Regarding the current research, the research on nitrogen content and protein content of crops are relatively mature (Xue et al., 2004; Zhu et al., 2007; Wang et al., 2012; Zheng et al., 2020), but the model accuracy of yield study still has more potential for improvement in practical production work. For example, Shen et al. (2009) used data assimilation method to predict rice yield based on radar image data with the root mean square error of 113 g·m^−2^. Yang et al. (2019) used unmanned aerial vehicle multispectral images to train a convolutional neural network model to predict rice yield with the root mean square error of 65.8 g·m^−2^. In the field of hyperspectral pre-processing, Yu et al. (2020a) used wavelet transform to pre-process unmanned aerial vehicle hyperspectral images and developed a nitrogen content estimation model in rice. Osco et al. (2020) used the first derivative transformation method to process the hyperspectral data to filter out the most appropriate wavelength to predict the nutrient content in orange leaves. Amirhossein et al. (2020) developed a yield estimation model for snap beans by using continuum removal. Yang et al. (2021b) used wavelet transform to remove hyperspectral noise and developed a model for estimating corn yield. He et al. (2018) estimated the canopy chlorophyll content of winter wheat based on the wavelet transform. Jin and Wang (2016) successfully traced the canopy transpiration of a desert plant by using first derivative spectra. Therefore, preprocessing crop canopy spectra with first derivative transformation, continuum removal, and wavelet transform is based on the certain study. However, few studies have used these preprocessing methods in combination, and this research attempts to use them in combination to be able to significantly improve the accuracy of the model.

On the other hand, most studies on canopy-level spectra adopted a single growth stage, usually the mature stage (Inoue et al., 1998; Zheng et al., 2016; Sampaio et al., 2018; Tuvdendorj et al., 2019; Yu et al., 2020b; Shao et al., 2021). Modeling studies based on the combination of multiple growth stages were not common. In fact, rice yield is a collective result of multiple growth stages. Relevant studies have revealed that rice yield was affected by various factors such as water, light, fertilizer, and quality. These impacts were exerted on every growth stage, exhibited as variations in growth, and eventually shown as differences in yield. Therefore, in addition to the spectral information of the maturity stage, the canopy spectral information of the important growth stages before the mature stage should also be included in the study to improve the yield estimation accuracy analysis. At present, there are two main methods for rice yield estimation using spectral data, statistical regression (Chang et al., 2005; Nguyen and Lee, 2006; Xue and Yang, 2008; Bajwa et al., 2010) and data assimilation (Huang et al., 2016; Xie et al., 2017; Mokhtari et al., 2018). Data assimilation could significantly improve the estimates of model parameters and model dynamic simulation ability to improve estimation accuracy (Reichle, 2008; Wang and Yu, 2021). However, this method requires the input of phenological characteristics, weather, soil, and variety coefficient, which are not easy to get and are complicated parameters. It seriously reduces the practical performance of the model, and the accuracy needs to be improved too (Wang et al., 2020). For example, Huang et al. (2015) collected weather and soil climate data and used convolutional neural network algorithm to build a winter wheat yield prediction model with an accuracy of 73.2 g·m^−2^. Ren et al. (2008) built a winter wheat yield prediction model based on Moderate-resolution Imaging Spectroradiometer (MODIS) products and used statistical regression method with an accuracy of 21.4 g·m^−2^. Therefore, in this study, two modeling methods, multiple stepwise regression and random forest, are chosen to compare the accuracy and finally select the best yield estimation model.

Therefore, this study selected four key stages of rice growth, the elongation stage, the heading stage, the flowering stage, and the milky stage, to study the impact of the combinations of spectral information of multiple growth stages on the yield prediction model.

## Materials and Methods

**Figure 1:**
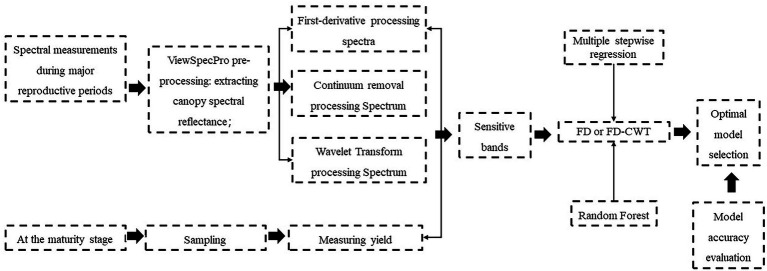
A flowchart of the research process.

As shown in Figure 1. This was a flowchart of the entire study, and the approach used had four main stages: data collection, data processing, model build, and model validation. A detailed description of the steps was given as follows.

### Experimental Design

Experimental site 1 was located in Yangzhou University experiment base, Jiangsu Province, China. The field experiment was a continuous experiment between 2015 and 2016, which was set up as three different experimental varieties (Nangeng 9,108, Yangnongdao No.1, and Yangdao No.6) with the same fertilizer variety for a total of 60 plots (N0: 0, N1: 100 kg.ha^−1^, N2: 200 kg.ha^−1^, N3: 300 kg.ha^−1^, N4: 400 kg.ha^−1^).

**Figure 2:**
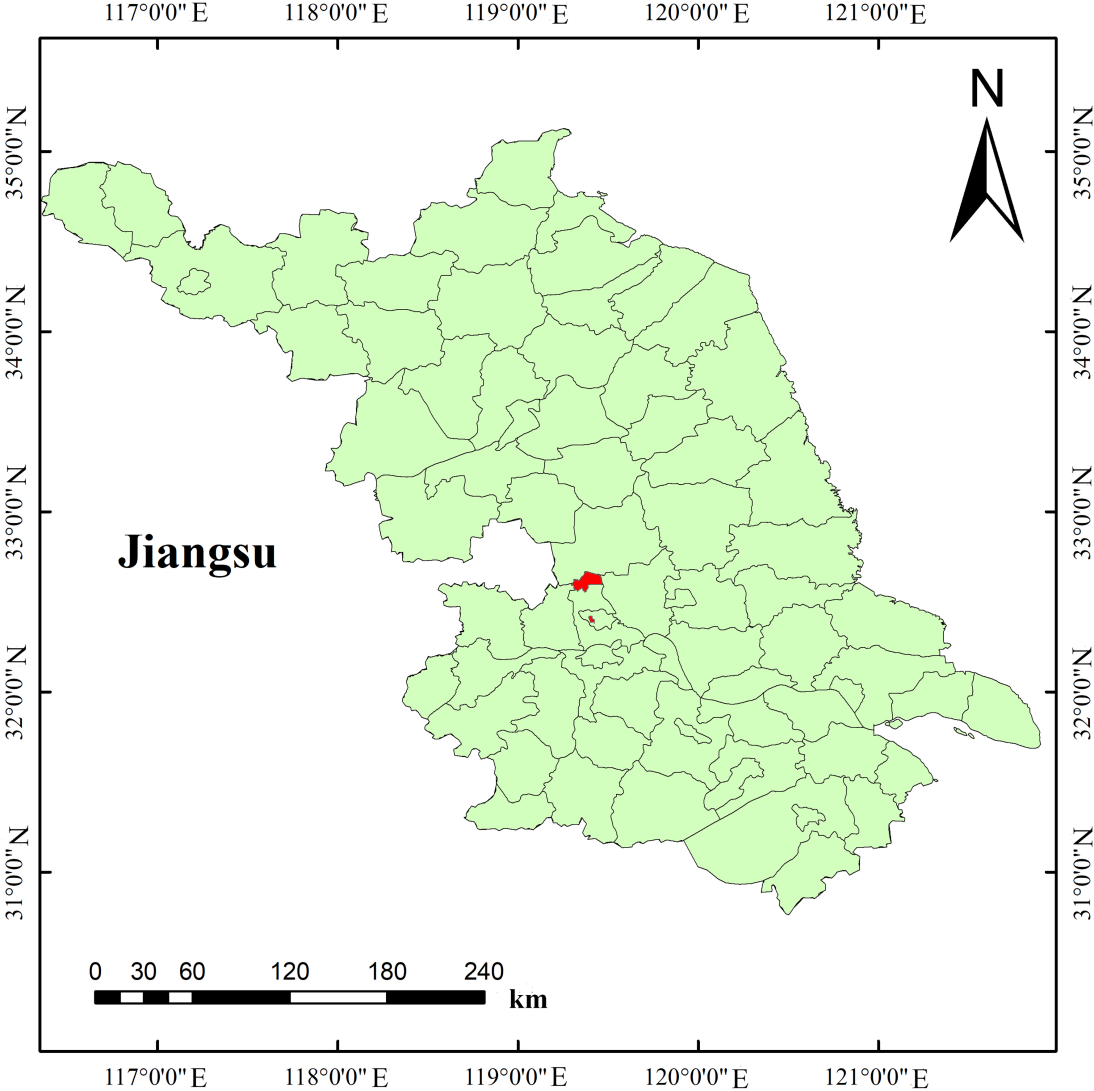
Experimental area overview.

**Figure 3:**
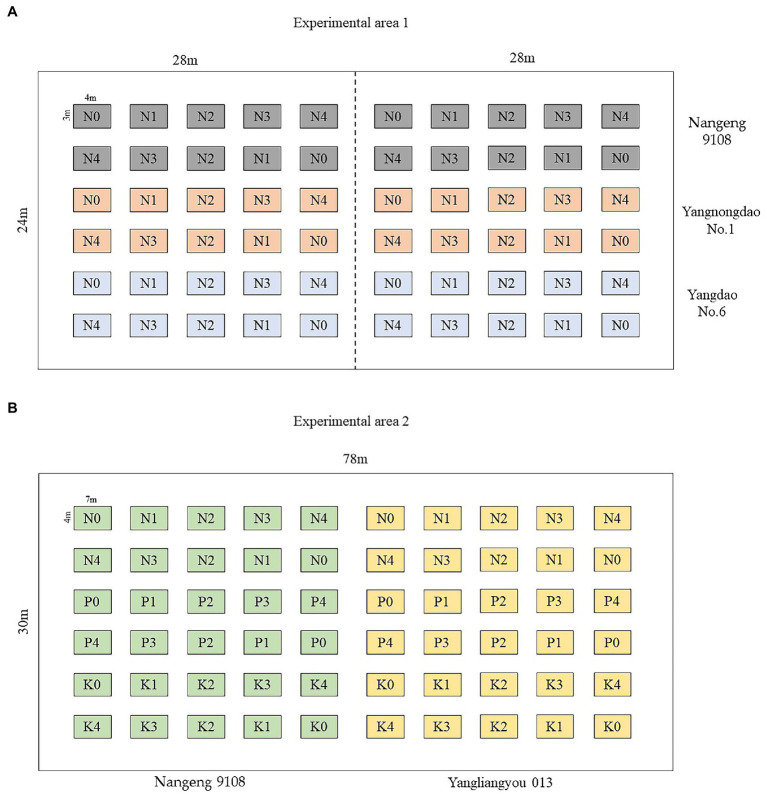
Experimental plot distribution map. **(A)** Experimental area 1, **(B)** Experimental area 2.

Experiment site 2 was set up at the test field in Gongdao Town, Yangzhou City, Jiangsu Province, China in 2019. The experiment was set up into a total of 60 plots of 2 rice variety (Nangeng 9,108, Yangliangyou 013), in which 5 N fertilizer levels (N0, N1, N2, N3, and N4) were set at 0, 100 kg.ha^−1^, 200 kg.ha^−1^, 300 kg.ha^−1^ and 400 kg.ha^−1^, 5 K fertilizer levels (K0, K1, K2, K3, and K4) were set at 0, 50 kg.ha^−1^, 100 kg.ha^−1^, 150 kg.ha^−1^ and 200 kg.ha^−1^, 5 P fertilizer levels (P0, P1, P2, P3, and P4) were set at 0, 100 kg.ha^−1^, 200 kg.ha^−1^, 300 kg.ha^−1^ and 400 kg.ha^−1^, respectively. Figure 2 shows the geographical location of the experimental area. Figure 3 is an experimental plot distribution map.

### Data Collection

#### Field Canopy Spectra Measurement

Measurements were made with a Fieldspec^®^3 (350–2,500 nm) Hi-Res spectrometer from ASD (Analytical Spectral Devices, Inc., CO, United States), with sampling intervals of 1.3 nm (in the 350–1,000 nm interval) and 2 nm (in the 1,000–2,500 nm interval). The spectra were measured in clear weather, without wind or with low wind speed, from 10:30 to 14:00 British Summer Time (BST). The probe was measured vertically downward at a distance of 0.6 m from the top of the plant crown, and the reflectance spectrum was the average of 10 repetitions within the plot (each measurement was made at a randomly selected location within the plot). The measurements were taken once at each of the four critical stages of rice, namely, elongation, heading, flowering, and milky, each measurement was calibrated by using a standard white reflectance panel (the standard white panel reflectance was 1).

#### Yield Determination

At the rice maturity stage, rice was harvested at a randomly selected 1 m^2^ area in each experimental field (avoiding the field edge). After harvest, the grains were threshed, sun-dried to a constant weight, and weighed to determine the rice yield of each experimental plot.

#### Spectral Variables

By reviewing the literature and results of related studies, it was found that spectral parameters such as red edge, yellow edge, and blue edge were frequently used in quality monitoring and prediction in the fields such as quality (Guo et al., 2019; Olivares Díaz et al., 2019; Yang et al., 2021a). Therefore, in this study, spectral characteristic parameters such as field canopy spectrum, first derivative spectrum of field canopy, four vegetation indices, and three edge parameters (red edge, blue edge, and yellow edge) were selected for parameter screening and model establishment, as listed in Table 1.

**Table 1:** Spectral variables.

Type		Symbol	Name	Definition	References
Vegetation index		NDVI	Normalized vegetation index	(NIR-R)/(NIR + R)	Huete, 1988
		DVI	Difference vegetation index	NIR-R	Gitelson et al., 2002
		RVI	Ratio vegetation index	R/NIR	Pearson and Miller, 1972
		EVI	Enhanced vegetation index	2.5(NIR-R)/(1 + NIR + 2.4R)	Jiang et al., 2008
Hyperspectral characteristic variable		λ_r_	Red edge position	Wavelength position of D_r_	Gong et al., 2002
	Red	D_r_	Red edge slope	First derivative spectral maximum within the red edge	Gong et al., 2002
	edge	SD_r_	Red edge area	Area of the first derivative spectrum within the red edge	Gong et al., 2002
		λ_b_	Blue edge position	Wavelength position of D_b_	Gong et al., 2002
	Blue	D_b_	Blue edge slope	First derivative spectral maximum within the blue edge	Gong et al., 2002
	edge	SD_b_	Blue edge area	Area of the first derivative spectrum within the blue edge	Gong et al., 2002
		λ_y_	Yellow edge position	Wavelength position of D_y_	Gong et al., 2002
	Yellow	D_y_	Yellow edge slope	First derivative spectral maximum within the yellow edge	Gong et al., 2002
	edge	SD_y_	Yellow edge area	Area of the first derivative spectrum within the yellow edge	Gong et al., 2002

NIR is any reflectivity in the wavelength range of 760–2,500 nm, R is any reflectivity in the wavelength range of 620–700 nm.

**Table 2:** Data characteristics.

Sample set	Number of samples	Minimum (g·m^−2^)	Maximum (g·m^−2^)	Mean value	Standard deviation	Variance	Coefficient of variation
Training	60	520	925	686.3	107.9	11633.7	0.16
Validation 1	60	450	925	659.6	102.1	10417.3	0.15
Validation 2	60	443	788	600.6	74.0	5469.7	0.12

### Hyperspectral Data Processing

#### Data Preprocessing

The hyperspectral data had large noise in the range of 350–2,500 nm. Therefore, the Savitsky-Golay (SG) filter (Savitzky and Golay, 1964) in Matlab 2016b version was used to smooth the original canopy reflectance spectrum, and the processed spectrum was marked as Original Reflectance (OR).

#### Conventional Spectral Transformation

To further eliminate the impact of noise and truly exhibit the spectral characteristics of ground objects, this study selected two conventional spectral transformation methods, first derivative transformation and continuum removal transformation of spectral reflectance. The spectrum obtained by first-derivative transformation after being pre-processed by the SG filter was noted as First-derivative (FD).

The spectrum obtained by continuum removal transformation after pre-processed by SG filter was noted as Continuum Removal (CR). The equation for calculation is as follows (Yang and Du, 2021).


$$S_{cr}=R/R_{C_{0}}$$

where *S_cr_* is the continuum removed spectral reflectance, *R* is the original spectral reflectance, and $R_{C_{0}}$ is the continuum linear reflectance.

#### Wavelet Transform

The property of wavelet transform is that time-domain features are added based on the Fourier transform. By decomposing the signals in time and frequency domains, wavelet transform achieves the separation and extraction of characteristic signals to obtain more effective information. Wavelet transforms are divided into two groups, continuous wavelet transform (CWT), and discrete wavelet transform (DWT). In this study, CWT was used to decompose the canopy reflectance spectral data at various scales. The equation for calculation is as follows.


$$W_{f}\left(a,b\right)=\overset{+\infty }{\underset{-\infty }{\int }}f\left(\lambda \right)\psi_{a,b}\left(\lambda \right)d\lambda$$

$$\psi_{a,b}\left(\lambda \right)=\frac{1}{\sqrt{a}}\psi \left(\frac{\lambda -b}{a}\right)$$

where ƒ(*λ*) is the spectral reflectance; *λ* is the number of spectral bands in the range of 350–2,500 nm; $\psi_{a,b}$ is the wavelet basis function; *a* is the scale factor; and *b* is the translation factor. The wavelet coefficient $W_{f}\left(a,b\right)$ contains two-dimensional data, the band and scale. The behavioral scales were generated and listed as the matrix of bands.

CWT on the rice canopy spectra was conducted in Matlab 2016b, and the 10 decomposition scales [1, 10] were set (Lamb et al., 2002), namely 2^1^, 2^2^, …, 2^10^. Correlation analysis was carried out between the transformation results under the 10 scales and rice yield, and the results were used to screen characteristic bands.

### Training Set and Validation Sets

The experimental data for 2015, 2016, and 2019 were selected, including the spectral data of the elongation stage, the heading stage, the flowering stage, and the milky stage. The sample size was 180. The samples from 2015 (*n* = 60) were used as the training set to establish a production estimation model. And the samples in 2016 (*n* = 60) and 2019 (*n* = 60) were, respectively, used as the validation set to verify the accuracy of the production estimation model. Table 2 shows the data characteristics.

### Model Building and Result Validation

The multivariate stepwise regression (MSR) method was used to establish a multiple linear regression model with multiple parameters. The central idea is to introduce independent variables one by one, on the condition of significantly improved coefficient of partial determination (partial R^2^) after introduction. At the same time, after introducing each new independent variable, the old independent variables should be tested one by one to remove those with insignificant partial R^2^. This process of introducing while removing was conducted until neither a new variable was introduced nor an old variable was removed. Its essence is to establish the “optimal” multiple linear regression equation. The equation for this type of model is (Uyanık and Güler, 2013):


$$y=b_{0}+b_{1}x_{1}+b_{2}x_{2}+b_{n}x_{n}+e$$

where y is the dependent variable, $x_{1},x_{2},\ldots ,x_{n}$ are the *n* independent variables used in the modeling, $b_{0},\;\;b_{1},b_{2},\ldots ,b_{n}$ are the constant terms corresponding to each independent variable, and e is the error term.

Random forest (RF) is a machine learning algorithm first proposed by Breiman. The algorithm uses the bootstrap resampling method to collect samples from the original sample and performs decision tree modeling for each sample extracted, combining them into multiple decision trees for prediction. The advantage of random forest is that the training is relatively fast and no cross-validation is required (Breiman, 2001). Therefore, random forest is widely used in the classification and prediction of remote sensing. When the random forest is applied to regression problems, the average of the results of each decision tree is the predicted value of the dependent variable.

The indicators selected for the model test were coefficient of determination (R^2^), root mean square error (RMSE), and mean absolute percentage error (MAPE).


$$R^{2}=\frac{\sum_{i=1}^{n}{\left(x_{i}-{\hat{x}}_{i}\right)}^{2}}{\sum_{i=1}^{n}{\left(x_{i}-\bar{x}\right)}^{2}}$$

$$RMSE=\sqrt{\frac{\sum_{i=1}^{n}{\left(x_{i}-{\hat{x}}_{i}\right)}^{2}}{n}}$$

$$MAPE=\frac{\sum_{i=1}^{n}\left|\frac{x_{i}-{\hat{x}}_{i}}{{\hat{x}}_{i}}\right|}{n}\times 100\%$$

Where n is the number of sample sets, $\bar{x}$ is the mean value of rice yield, $x_{i}$ is the measured value of rice yield, and ${\hat{x}}_{i}$ is the predicted value of the model.

The higher the value of R^2^, the better the goodness of fit of the corresponding model. RMSE and MAPE tell how accurate the predictions are, and they are two indicators evaluating the regression model. The smaller the values of RMSE and MAPE, the more accurate the model predicts.

## Results and Analysis

### Analysis of Canopy Spectral Transformation of Rice in Various Growth Stages

**Figure 4:**
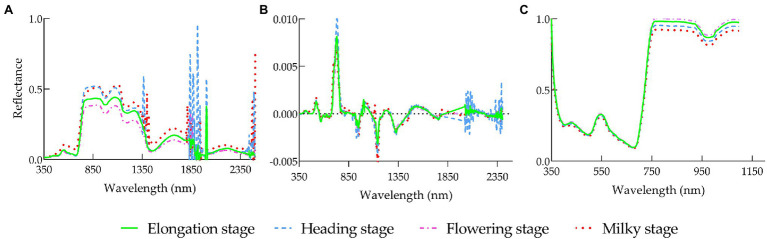
Spectral reflectance of rice leaf canopy under different treatments in various growth stages: **(A)** OR, **(B)** FD, **(C)** CR.

**Figure 5:**
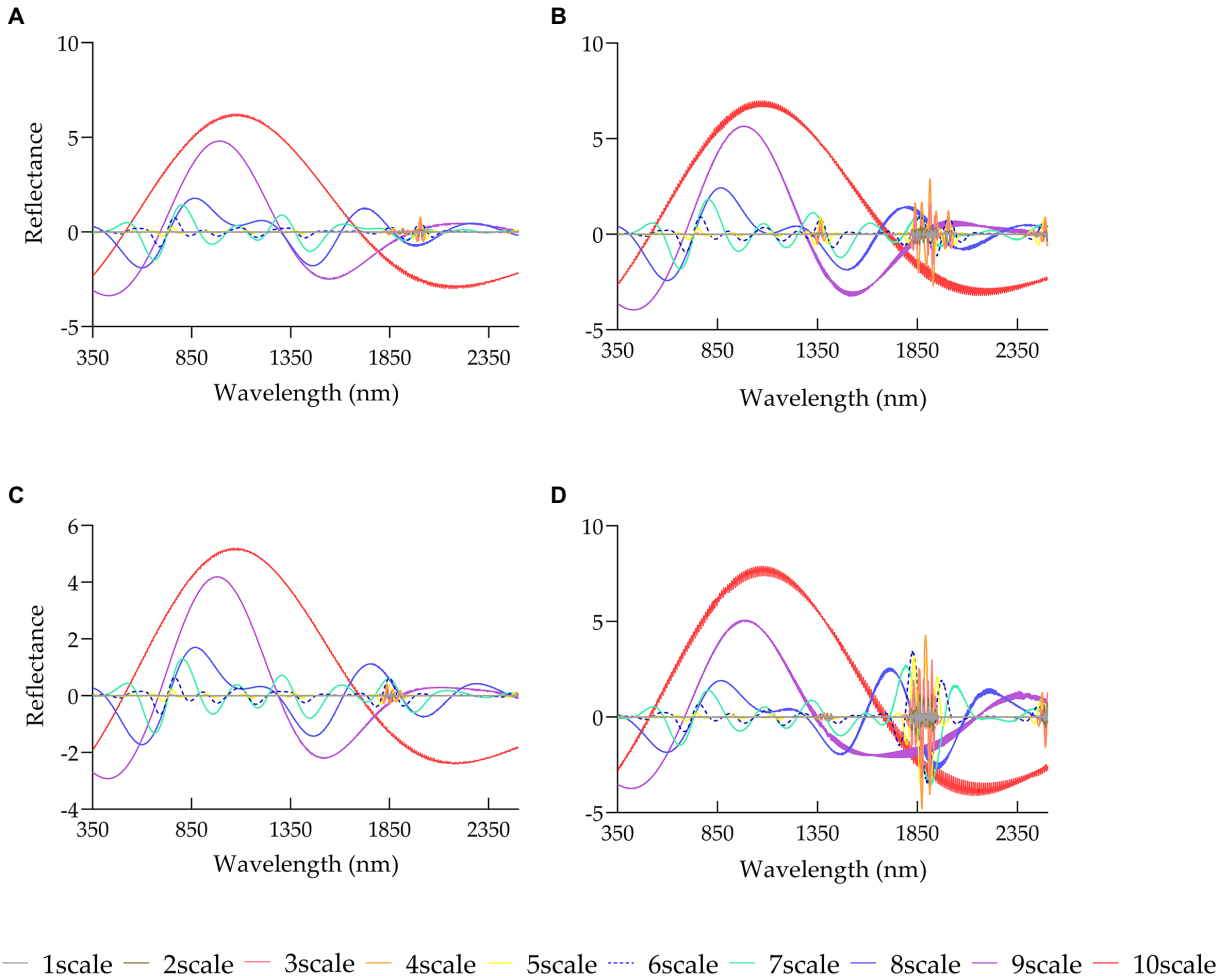
Changes in reflectance of wavelet transform of rice canopy spectra in various growth stages: **(A)** elongation stage, **(B)** heading stage, **(C)** flowering stage, **(D)** milky stage.

The first derivative, continuum removal, and wavelet transform were performed on the original reflectance. All three methods showed varied curves from the pattern of OR (Figures 4 5). As shown in Figure 4, the reflectance of OR and CR was quite different in the four stages in the range of 800–1,100 nm, whereas it was difficult for the FD treatment to intuitively show the difference in spectral reflectance in various stages. Figure 5 shows the patterns of rice canopy spectra for 10 scales of transformations at various stages. It can be seen from Figure 5 that the patterns of the four stages were relatively flat on scale [1, 5], with no clear spectral features, were all wave-shaped on scale [6, 8], turned to parabolic on scale [9, 10], and were approaching a straight line beyond 2000 nm. Overall, the spectral features were more distinct than the original spectrum after being transformed at scale 6, 7, 8, 9, and 10.

### Correlation Analysis

#### Correlation Between Rice Yield and Conventional Spectral Transformations

**Figure 6:**
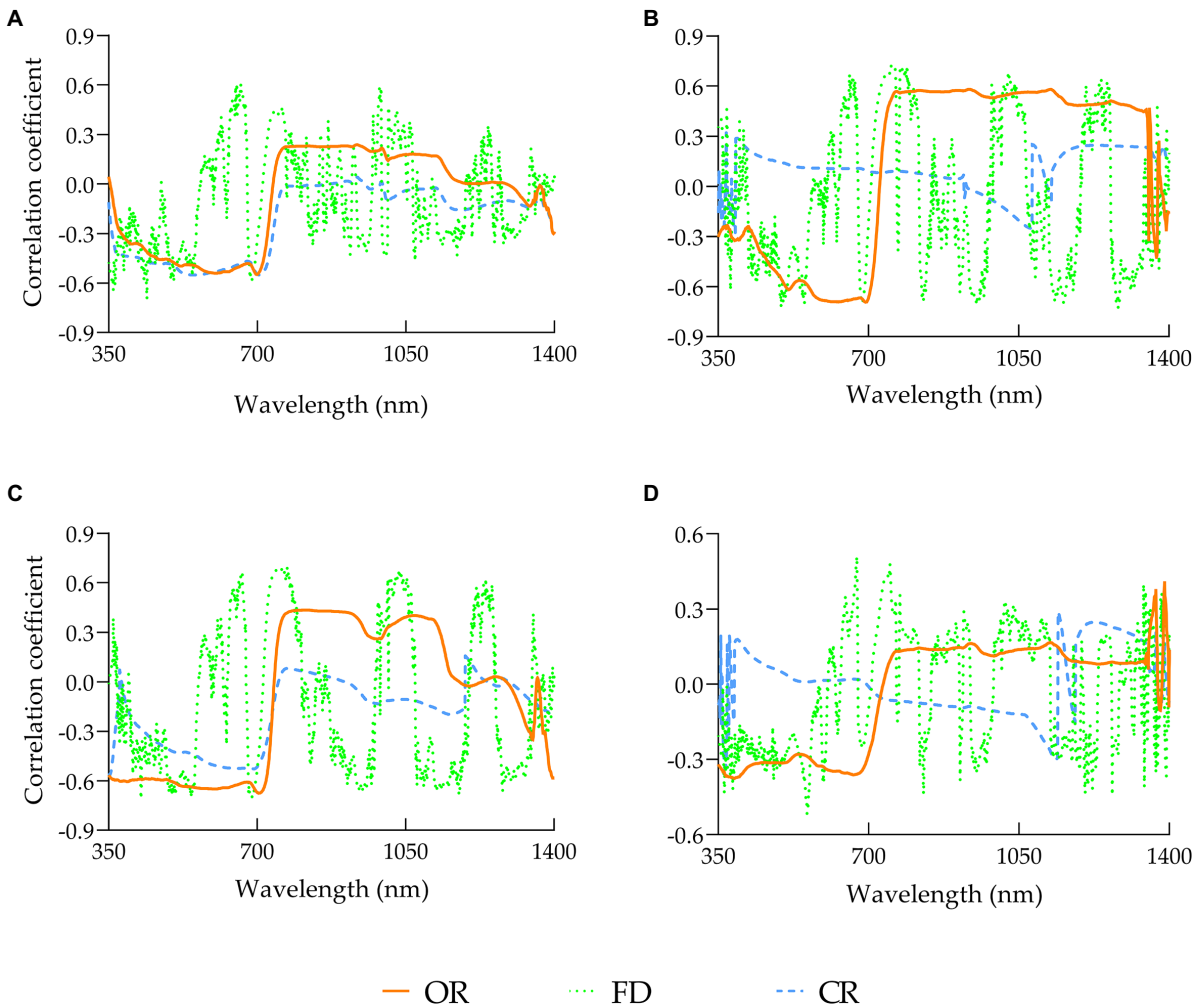
Correlation between rice yield and conventional spectral transformations in various growth stages: **(A)** elongation stage, **(B)** heading stage, **(C)** flowering stage, **(D)** milky stage.

To further take advantage of the rice canopy spectra to predict the rice yield, based on the correlation analysis between the original reflectance and rice yield, this study also conducted a correlation analysis between the first derivative spectra and rice yield, and between the reflectance spectra after continuum removal and rice yield (Figure 6). It can be seen that the original spectrum at the jointing stage was significantly correlated with the yield in the range of 400–720 nm. After the first derivative of the spectrum was processed, most of the sensitive bands were still retained in the visible light range, in addition, the range for sensitive bands selection was extended to the near-infrared region beyond 800 nm, such as 910–925 nm, 935–966 nm, 983–1,010 nm, etc. At the heading stage, the strongest correlation after the first-derivative treatment increased to r^′^_1283_ nm = −0.73 from r_694_ nm = −0.69 in the original spectrum. The result of the flowering stage was similar that the strongest correlation increased from r_705_ nm = −0.67 to r^′^_686_ nm = −0.71. Compared with the previous three stages, the sensitive bands of the milky stage were narrower, and overall, the correlation was decreased as well. Considering the correlation performance of the three treatments, FD > OR > CR, therefore, the spectral spectrum after FD treatment was selected as an independent variable to be introduced into the yield prediction model.

### Correlation Between Rice Yield and Wavelet Transform

**Figure 7:**
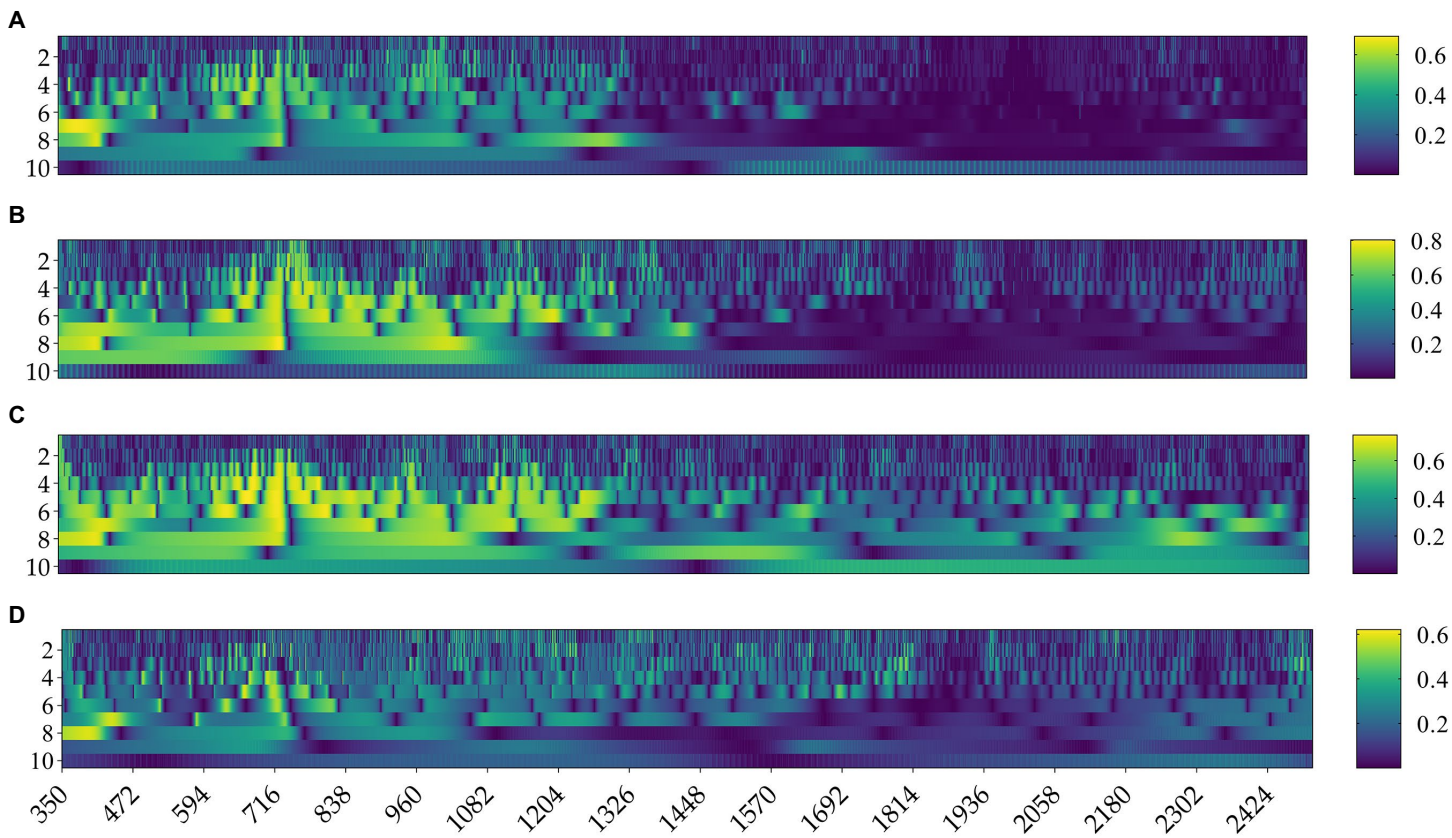
Absolute value of correlation coefficients of different wavelet coefficients with rice yield in various growth stages: **(A)** elongation stage, **(B)** heading stage, **(C)** flowering stage, **(D)** milky stage.

Figure 7 shows the correlation coefficient matrix of rice yield and rice canopy spectra after 10-dimensional CWT at various stages. As shown in Figure 7, the sensitive bands related to rice yield mainly focused on the decomposition at scale [4, 9], and the correlations were weak at scale [1, 3] and [10]. The result after the wavelet transform was compared with the result after the first derivative transform. It was shown that at the elongation stage, the maximum correlation coefficient r appeared at scale [4] at 683 nm with a value of 0.74, significantly higher than the maximum *r*-value of 0.64 at 440 nm of first derivative transform. At the heading stage, the maximum correlation coefficient r was at scale [8] at 732 nm with a value of 0.81, higher than the maximum correlation of −0.73 at 1283 nm of the first derivative transform. At the flowering stage, the maximum correlation coefficient was 0.74 at scale [5] at 675 nm, slightly higher than the maximum correlation of −0.71 at 426 nm of the first-derivative. At the milky stage, the maximum correlation coefficient was 0.65 at scale [4] at 570 nm, significantly higher than the maximum correlation of −0.51 at 557 nm of the first derivative transform. In addition, the number of sensitive bands of spectral reflectance to rice yield under the first derivative treatment was significantly less than that treated by CWT. Therefore, the overall results demonstrated that CWT was significantly better than FD. The effective spectral signals were better displayed after wavelet transform, and it was conducive to digging into the information to facilitate subsequent research and analysis.

To explore which scales of the wavelet transform have the most potential in retrieving rice yield, the 10 dimensions under each growth stage were analyzed independently (Supplementary Figure 1). The results showed that at the elongation stage, the absolute values of the correlation coefficients between spectral reflectance and rice yield under wavelet transform at scale [4, 6] and [8] were increased significantly, and the number of sensitive bands also increased substantially compared with the FD transform. The improvement by wavelet transform was more distinct at the heading and flowering stages, with dominant scales concentrated in [4, 9]. While the milky stage was significantly improved at scales [4, 5] and [7, 8]. Therefore, the overall results indicated that the wavelet transform of the original spectrum had strong adaptability at scale [8], and performed well across all the four growth stages.

### Correlation Between Vegetation Indices and Rice Yield

Four vegetation index (VI), Normalized Difference Vegetation Index (NDVI), Ratio Vegetation Index (RVI), Difference Vegetation Index (DVI), and Enhanced Vegetation Index (EVI), were calculated using the red and near-infrared bands, and the correlation between each of them with measured yield was analyzed. The results are shown in Table 3. It is exhibited that the Pearson correlation coefficients of the four vegetation indices all increased first and then decreased as the growth stage advanced and peaked at the heading stage. Therefore, it is speculated that the vegetation indices at the heading and flowering stages would perform better for the prediction of rice yield in the later stage.

**Table 3:** Summary of optimal parameters of vegetation index.

Stage	RVI	NDVI	DVI	EVI
	Correlation coefficient	Correlation coefficient	Correlation coefficient	Correlation coefficient
Elongation stage	0.58	0.59	0.34	0.43
Heading stage	0.74	0.73	0.62	0.67
Flowering stage	0.72	0.71	0.56	0.64
Milky stage	0.61	0.63	0.24	0.34

### Correlation Between FD-Based Hyperspectral Characteristic Parameters and Rice Yield

The characteristic parameters such as red edge, yellow edge, and blue edge were calculated, and further, they were normalized and differentially calculated. The correlation analysis was carried out between the obtained values and rice yield, and the results are shown in Table 4. As shown, the correlation coefficients between the hyperspectral parameters and yield at the heading and flowering stages were generally higher than those at the elongation and milky stages. And the three hyperspectral parameters, λ_r_, SD_r_/SD_b_, and (SD_r_ − SD_b_)/(SD_r_ + SD_b_), performed best regarding adaptation and were strongly correlated with the yield at all four stages.

**Table 4:** Correlation analysis between spectral characteristics variable and rice yield at different stages.

Hyperspectral characteristic	Correlation coefficient			
variable	Elongation stage	Heading stage	Flowering stage	Milky stage
λ_r_	0.59	0.66	0.71	0.61
λ_b_	−0.12	0.23	0.30	0.07
λ_y_	0.12	0.18	0.01	0.45
D_r_	0.34	0.61	0.58	0.21
D_b_	−0.39	−0.40	−0.58	−0.21
D_y_	−0.12	−0.21	−0.03	−0.38
SD_r_	0.28	0.58	0.51	0.22
SD_b_	−0.47	−0.54	−0.63	−0.25
SD_y_	−0.14	−0.03	−0.20	0.11
SD_r_ − SD_b_	0.32	0.61	0.56	0.26
SD_r_ − SD_y_	0.30	0.60	0.54	0.22
SD_b_ − Sd_y_	−0.45	−0.67	−0.60	−0.40
SD_r_/SD_b_	0.58	0.73	0.72	0.63
SD_r_/SD_y_	0.52	0.72	0.42	−0.17
SD_b_/SD_y_	−0.39	−0.62	−0.46	−0.39
(SD_r_ − SD_b_)/(SD_r_ + SD_b_)	0.64	0.73	0.72	0.67
(SD_r_ − SD_y_)/(SD_r_ + SD_y_)	0.52	0.73	0.51	0.11

λ_b_ is blue edge position, D_b_ is blue edge slope, SD_b_ is blue edge area; λ_y_ is yellow edge position, D_y_ is yellow edge slope, SD_y_ is yellow edge area; λ_r_ is red edge position, D_r_ is red edge slope, SD_r_ is red edge area.

### Construction of Rice Yield Prediction Models

The yield of rice is a collective result of multiple growth stages, and each growth contributed spectral variables that are closely related to yield. Therefore, this study combined the spectral variables of multiple growth stages to predict rice yield and adopted MSR and RF to establish prediction models to determine the optimal combination of growth stages. In addition, to verify whether the wavelet transform could improve the prediction accuracy of the yield estimation model, two prediction models were established in this study. One was the yield prediction model based on the first derivative transform, the other one was based on first derivative-wavelet transform.

### Comparison of Different Models Based on First Derivative Transform

The MSR and RF models established based on the first derivative transformation of multiple growth stages are shown in Table 5. As far as the results of a single growth stage were concerned, the model of the heading stage performed the best. The optimal MSR models at the corresponding four growth stages were the heading stage model, the heading-milky stage model, the elongation-heading-milky stage model, and the elongation-heading-flowering-milky stage model, respectively. The optimal RF models were the heading stage model, the heading-flowering model, the elongation-heading-milky model, the elongation-heading-flowering-milky model.

**Table 5:** Rice yield prediction model based on first derivative transform.

Stage combination	MSR			RF		
	R^2^	RMSE (g·m^−2^)	MAPE (%)	R^2^	RMSE (g·m^−2^)	MAPE (%)
Elongation	0.46	92.50	11.80	0.49	83.70	8.70
Heading	0.50	67.60	7.50	0.55	63.10	7.10
Flowering	0.44	72.50	8.40	0.46	75.00	8.20
Milky	0.49	80.40	8.30	0.51	74.50	8.10
Elongation-heading	0.48	73.80	8.30	0.50	65.50	7.70
Elongation-flowering	0.50	67.20	8.50	0.55	63.90	8.00
Elongation-milky	0.50	70.10	8.10	0.61	60.90	7.20
Heading-flowering	0.51	69.80	7.50	0.59	60.40	6.90
Heading-milky	0.55	64.10	7.10	0.50	69.50	7.60
Flowering-milky	0.49	66.50	7.40	0.53	63.30	8.10
Elongation-heading-flowering	0.60	63.60	7.70	0.62	57.60	6.80
Elongation-heading-milky	0.62	58.30	6.90	0.67	52.60	6.50
Elongation-flowering-milky	0.54	65.70	7.80	0.55	60.50	7.50
Heading-flowering-milky	0.58	63.50	7.10	0.60	55.50	6.60
Elongation-heading-flowering-milky	0.70	50.30	5.60	0.77	45.10	5.50

### Comparison of Different Models Based on First Derivative-Wavelet Transform

The MSR models and RF models established based on first derivative-wavelet transform of multiple growth stages are shown in Table 6. Regarding the modeling results of a single growth stage, the model of the heading stage performed the best. Comparing the models of each growth stage, it can be seen that the optimal MSR models for the corresponding four growth stages were the heading stage model, the heading-flowering stage model, the elongation-heading-flowering stage model, and the elongation-heading-flowering-milky model, respectively. The optimal RF models were the heading stage, the heading-milky stage, the elongation-heading-milky stage, the elongation-heading-flowering-milky stage.

**Table 6:** Rice yield prediction model based on first derivative-wavelet transform.

Stage combination	MSR			RF		
	R^2^	RMSE (g·m^−2^)	MAPE (%)	R^2^	RMSE (g·m^−2^)	MAPE (%)
Elongation	0.54	88.30	10.20	0.50	76.60	9.90
Heading	0.64	60.90	7.90	0.66	58.00	7.80
Flowering	0.56	64.50	8.20	0.58	66.40	7.90
Milky	0.48	72.40	9.00	0.53	69.90	8.70
Elongation-heading	0.65	58.10	7.30	0.67	58.30	6.70
Elongation-flowering	0.60	65.40	8.70	0.60	60.80	6.90
Elongation-milky	0.66	60.90	8.10	0.61	60.20	6.50
Heading-flowering	0.68	57.50	7.00	0.62	57.20	6.60
Heading-milky	0.65	59.60	7.80	0.69	54.60	6.20
Flowering-milky	0.60	61.30	7.90	0.65	54.80	6.70
Elongation-heading-flowering	0.73	47.10	6.50	0.74	47.40	5.90
Elongation-heading-milky	0.67	50.30	6.80	0.75	46.00	5.70
Elongation-flowering-milky	0.66	54.70	7.20	0.71	58.40	6.40
Heading-flowering-milky	0.68	48.30	6.60	0.73	50.90	6.00
Elongation-heading-flowering-milky	0.81	37.60	4.80	0.86	35.50	4.60

A comprehensive comparison of Tables 5 6 showed that the most suitable growth stage combinations for rice yield estimation was the elongation-heading-flowering-milky stage. In the model validation section, this study validated the MSR model and RF model for the four stages combinations.

### Validation of the Predictive Model

The evaluation results of the two modeling methods based on validation set 1 were shown in the table (Table 7). VI was combined with FD and FD-CWT, respectively, for a comparative analysis of the two modeling approaches. For the MSR model, the combination of VI with CWT-FD improved the modeling set R^2^ by 0.11 and reduced the RMSE and MAPE by 12.70 g·m^−2^ and 0.80%, respectively, while the validation set R^2^ improved by 0.11 and reduced the RMSE and MAPE by 15.5 g·m^−2^ and 1.4%, respectively. For the RF model, the modeling set R^2^ improved by 0.09 and RMSE and MAPE decreased by 9.60 g·m^−2^ and 1.00%, respectively, and the validation set R^2^ improved by 0.05 and RMSE and MAPE decreased by 11.80 g·m^−2^ and 0.70%, respectively. Consequently, the most suitable combination of independent variables for estimating rice yield was VI-FD-CWT. In terms of the effect of different modeling algorithms, the RF algorithm gave the best results with modeling sets R^2^, RMSE, and MAPE of 0.86, 35.50 g·m^−2^, and 4.60%, respectively, and validation sets R^2^, RMSE, and MAPE of 0.85, 33.40 g·m^−2^, and 4.30%, respectively. Based on the four growth stages and CWT-FD-VI combination, the RF model was the best estimation model for rice yield.

**Table 7:** Comparison of the two modeling approaches.

			Training set			Validation set		
Stage combination	Model algorithm	Independent variable	R^2^	RMSE (g·m^−2^)	MAPE (%)	R^2^	RMSE (g·m^−2^)	MAPE (%)
Elongation-	MSR	FD-VI	0.70	50.30	5.60	0.68	51.80	6.10
Heading-		CWT-FD-VI	0.81	37.60	4.80	0.77	36.30	4.70
Flowering-	RF	FD-VI	0.77	45.10	5.60	0.80	45.20	5.00
Milky		CWT-FD-VI	0.86	35.50	4.60	0.85	33.40	4.30

The optimal MSR and RF models generated by the two transformation methods based on combinations of four growth stages were tested. The validation sets were independent sample sets, and the results are shown in Supplementary Figures 2, 3.

To verify whether different varieties and nitrogen fertilizer levels affect the prediction accuracy of the models, completely independent validation sets were used in this study to re-evaluate the optimal MSR model and RF model. The evaluation results are shown in Supplementary Figure 4. The validation results of the validation set 2 showed that the RF model was superior to the MSR model, with R^2^ improving by 0.08 and RMSE and MAPE decreasing by 6.3 g·m^−2^ and 1.3%, respectively.

## Discussion

Literature and previous studies have already proved that the spectral reflectance can tell the growth status of crops to various extents. However, the existing models are usually established based on the original spectrum without any processing, leaving a lot of room for improvement regarding the model accuracy. For example, Li et al. used a successive projection algorithm (SPA) to determine characteristic bands and then established an estimation model for estimating the pH of water body (Li and Guo, 2021). The spectral preprocessing methods such as first derivative and continuum removal which have been commonly used in recent years could amplify the effective information in the spectrum to a certain extent. For example, Yuan et al. used SG to smooth the hyperspectral data of the original spectrum, screened the sensitive bands, and identified the early rice blast disease with an accuracy of 90% (Yuan et al., 2021). Gao et al. adopted the first derivative and continuum removal in the estimation of the phosphorus content of grassland forages and pointed out that the first derivative was the most effective spectral preprocessing method (Gao et al., 2019). The range of characteristic spectral bands after processing by first derivative could be extended to the infrared region. This conclusion is consistent with the previous findings. Previous research of our lab revealed that the field rice canopy spectrum was the collective result of multiple factors including weather and rice variety. In addition, noise was also introduced into the canopy spectral data collected in the field due to human reasons and the machine itself. It is difficult for the conventional spectral preprocessing methods to deep excavate effective information. Therefore, in this study, the original spectrum after SG smoothing was taken and subjected to continuous wavelet transform to eliminate spectral noise. The results demonstrated that the wavelet transform of original spectrum could not only greatly boost its correlation with rice yield, but also increase the number of sensitive bands in various stages compared with the first derivative transform, with an especially distinct effect in the flowering and heading stages. At the same time, the comparative analysis also revealed that the wavelet transform under scale [8] was the most effective for mining effective information, and its strong ability was seen for all the four stages, basically consistent with the previous research results (Li et al., 2019; Zhou et al., 2021). Therefore, wavelet transform can be used in the next step of research to establish estimation models for important agronomic parameters in each growth stage.

In terms of hyperspectral parameter selection, correlation analysis showed that various parameters demonstrated different sensitivities in different growth stages. The parameters NDVI, RVI, and “tri-edge” parameters all performed nicely in all the four growth stages after difference, ratio, or normalization transformations. However, the correlations between them with rice yield were generally higher in the heading and flowering stags than in the other two growth stages. By analyzing the sensitive bands selected by various hyperspectral studies in recent years (Wu and Shi, 2004; Xie et al., 2014; Bagchi et al., 2016), it was found that most of them were in the near-infrared region, and there have been few related applications in the field of visible light. Nevertheless, in our study on the bands selected by the optimal models for different growth stages, it was shown that except for the red-edge parameter, all the others were distributed in the visible light range. The results of the present study demonstrated that the established prediction models based on wavelet transform could greatly reduce the difficulty of parameter acquisition and improve the practical model performance. The comparison of previous studies showed that the established prediction model for yield was often limited to using a single vegetation index. For example, Lai et al. used NDVI at the mature stage to build a rice panicle differentiation prediction model (Lai and Lin, 2021). Nazir et al. used Sentinel-2 satellite images together with different single vegetation index to predict rice yield (Nazir et al., 2021). However, usually, this method had low accuracy, and in practical applications, issues such as overfitting were seen. Huang et al. pointed out that such disadvantages existed when simply using the relationship between vegetation index and crop yield to build a model (Huang et al., 2019). In addition, in the optimal models regarding combinations of different growth stages, the four vegetation indices checked in the study were not included in the final optimal model. It indicated that these four vegetation indices cannot be used to accurately estimate the yield of rice. In the next stage of research, we may consider replacing them with other vegetation indices, such as Soil-Adjusted Vegetation Index (SAVI), Optimized Soil-Adjusted Vegetation Index (OSAVI), Green Normalized Difference Vegetation Index (GNDVI), Normalized Difference Water Index (NDWI), etc.

In terms of growth stage selection, our study found that the heading and flowering stages were the best predictors of rice yield, followed by the jointing and milky stages. The trend was not a monotonically increasing curve following the growth stages, but a parabolic curve that first increased and then decreased. Presumably, it may be because of the strong interference of soil and weeds due to the low coverage rate of rice before the jointing stage. In addition, the nutrient accumulation of rice in the booting stage has not finished yet, and the spectral change is mainly affected by the growth of stems and leaves. Therefore, the spectral information of rice at the early growth stages was not suitable for yield estimation. The heading and flowering stages of rice were the key stages to yield. Gradually, rice transitioned from nutritional phase to reproductive phase, and the crop population was coordinated. Therefore, the hyperspectral information of these two stages contributed the most to the rice yield estimation model. Most of the current studies on rice yield were based on remote-sensing information of a single growth stage. For example, Jin et al. established a winter wheat yield estimation model using a combination of multiple vegetation indices at the heading stage and gave a verification R^2^ of 0.69, but they did not explore much information on the growth stages (Jin et al., 2022). Therefore, the present study comprehensively utilized the information on multiple growth stages based on previous studies to verify and further explore the significance and role of the spectra of different combinations of growth stages on the rice yield prediction model.

The research results already demonstrated that the accuracies of the regression models based on the combinations of multiple growth stages were higher than those established by the parameters of single growth stages. Therefore, the introduction of information on multiple growth stages may significantly improve the accuracy of the prediction model. The optimal combination of growth stages was elongation-heading-flowering-milky. In addition, two validation sets were set up in this study considering the influence of variety and fertilizer variety on the accuracy of the model. In this study, to verify the generality of the optimal growth stage combination model, another validation set using a different variety and a different fertilizer test were used to verify the accuracy of the model. The R^2^ of the MSR model decreased by 0.05 and the RMSE and MAPE increased by 7.40 g·m^−2^ and 1.2%, respectively. The R^2^ of the RF model decreased by 0.05 and the RMSE and MAPE increased by 4.00 g·m^−2^ and 0.3%, respectively. Therefore, it proved that the generalizability of the RF model was higher than the MSR model.

At present, the research on estimating rice yield still faces many challenges, and more exploration is urgently needed. First, the hyperspectral prediction model has been applied in various fields in recent years, but its mechanism investigation remains insufficient. For example, the technology still cannot distinguish different varieties by spectrum. At present, most of the models were derived from empirical models. With the continuous advancement of science and technology, hyperspectral technology become more and more mature in the future. Secondly, with the continuous innovation in machine learning field in recent years, more and more algorithms have been applied to the field of agricultural remote sensing, such as the Support Vector Machine algorithm, Gaussian Process Regression algorithm, etc. Appropriate algorithms can significantly improve the accuracy of the prediction model and are a great help to practicability improvement. In addition, the full rise of agricultural drones will provide new directions for large-scale yield estimation too.

## Conclusion

By comprehensive analysis and comparison of correlations and modeling, it was demonstrated that wavelet transform was the most effective spectral preprocessing method, followed by first-derivative. This study found that after the original spectrum was processed by the first-derivative and wavelet transform, the effective information was amplified and enhanced, and the ability to characterize rice yield became stronger. Therefore, the wavelet transform and first derivative transform methods have important application values in enhancing spectral characteristics. Secondly, the rice yield prediction models established based on combining multiple growth stages could significantly improve the prediction accuracy. The RF model established by combining first derivative-wavelet transform and the four growth stages (elongation-heading-flowering-milky) carried out the best prediction, with modeling set R^2^ of 0.86, RMSE of 35.50 g·m^−2^, and MAPE of 4.60%. The validation set 1 had the results as R^2^ of 0.85, RMSE of 33.40 g·m^−2^, and MAPE of 4.30%. The validation set 2 had the results as R^2^ of 0.80, RMSE of 37.40 g·m^−2^, and MAPE of 4.60%.

## Data Availability Statement

The raw data supporting the conclusions of this article will be made available by the authors, without undue reservation.

## Author Contributions

CG and CT: conceptualization and formal analysis. CT: supervision. CG and SJ: data curation and validation. CG: prepared and revised the manuscript. SJ, QH, WL, RZ, ZZ and ZH: provided technical support. CG, WM, HZ, BL, XX: visualization and original draft. All authors contributed to the article and approved the submitted version.

## Funding

This study was supported by the National Natural Science Foundation of China (32071902), the Key Research Program of Jiangsu Province, China (BE2020319), the Yangzhou University Interdisciplinary Research Foundation for Crop Science Discipline of Targeted Support (yzuxk202007 and yzuxk202008), and the Priority Academic Program Development of Jiangsu Higher Education Institutions (PAPD).

## Conflict of Interest

The authors declare that the research was conducted in the absence of any commercial or financial relationships that could be construed as a potential conflict of interest.

## Publisher’s Note

All claims expressed in this article are solely those of the authors and do not necessarily represent those of their affiliated organizations, or those of the publisher, the editors and the reviewers. Any product that may be evaluated in this article, or claim that may be made by its manufacturer, is not guaranteed or endorsed by the publisher.

## Supplementary Material

The Supplementary Material for this article can be found online at: https://www.frontiersin.org/articles/10.3389/fpls.2022.931789/full#supplementary-material

Supplementary Figure 1 Comparison of the number of sensitive bands after wavelet transform at different scales in various growth stages: **(A)** elongation stage, **(B)** heading stage, **(C)** flowering stage, **(D)** milky stage.

Supplementary Figure 2 Test results of four growth stages combination model based on first derivative transform: **(A)** MSR model, **(B)** RF model.

Supplementary Figure 3 Test results of four growth stages combination model based on first derivative-wavelet transform: **(A)** MSR model, **(B)** RF model.

Supplementary Figure 4 Model test results based on the validation set 2: **(A)** MSR model, **(B)** RF model.
