# Supplementary figures and images for: Rice Yield Estimation Based on Continuous Wavelet Transform With Multiple Growth Periods
Source: Front Plant Sci. 2022 Jul 1;13:931789. doi: 10.3389/fpls.2022.931789 (PMC9285008; doi:10.3389/fpls.2022.931789)

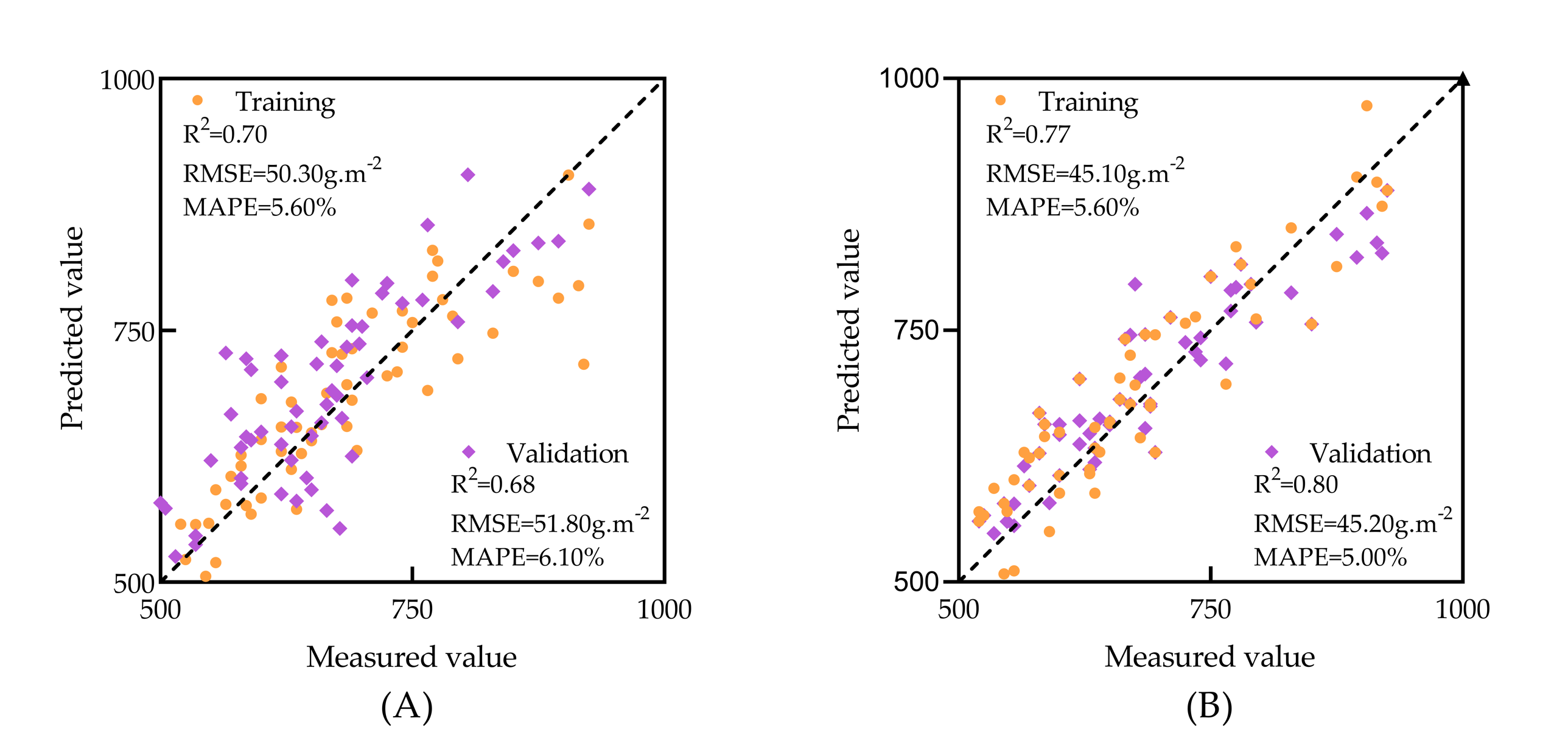

Supplement: Supplementary Figure 2 — Test results of four growth stages combination model based on first derivative transform: (A) MSR model, (B) RF model. [file Image_2.TIF]

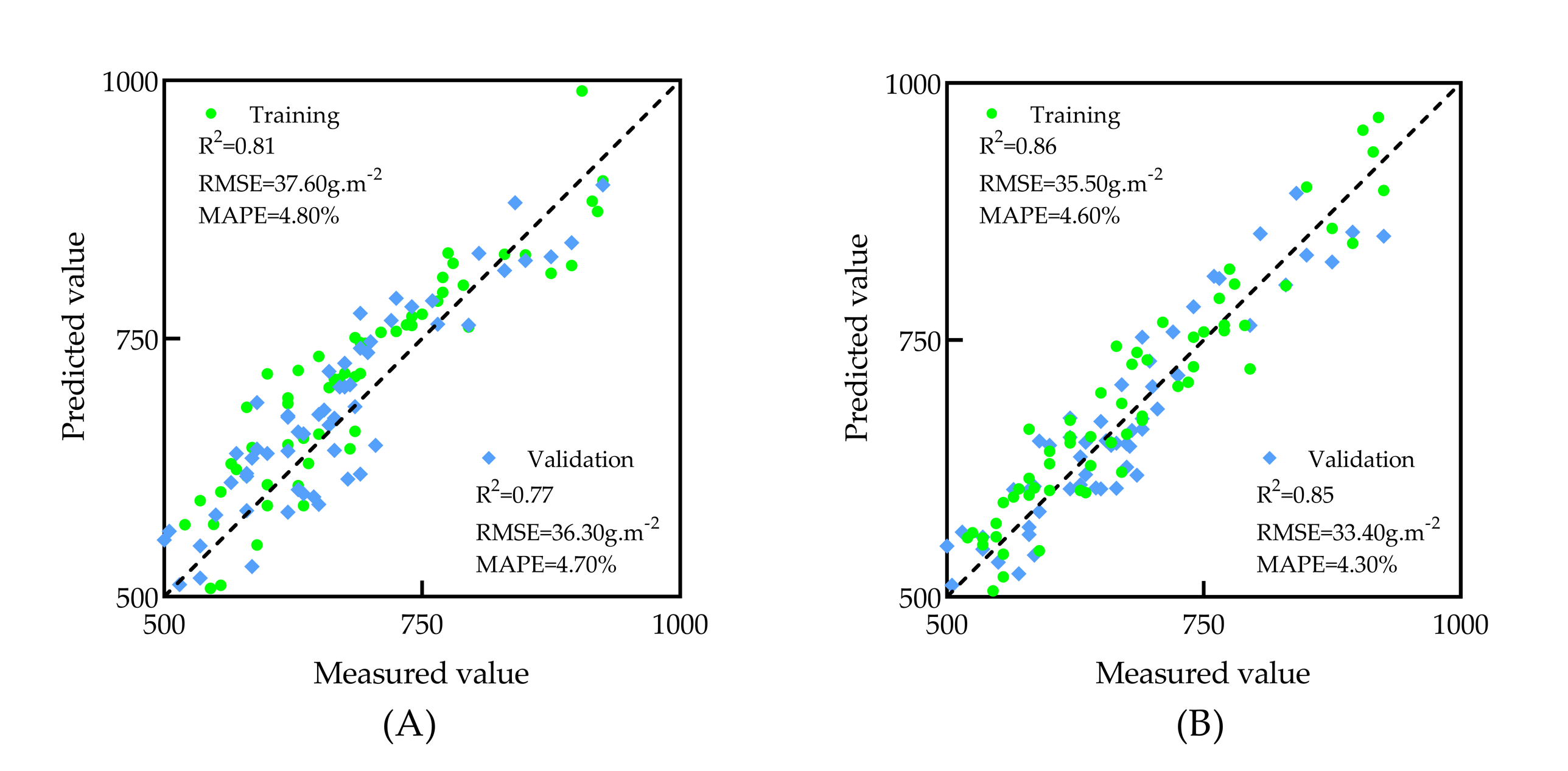

Supplement: Supplementary Figure 3 — Test results of four growth stages combination model based on first derivative-wavelet transform: (A) MSR model, (B) RF model. [file Image_3.TIF]

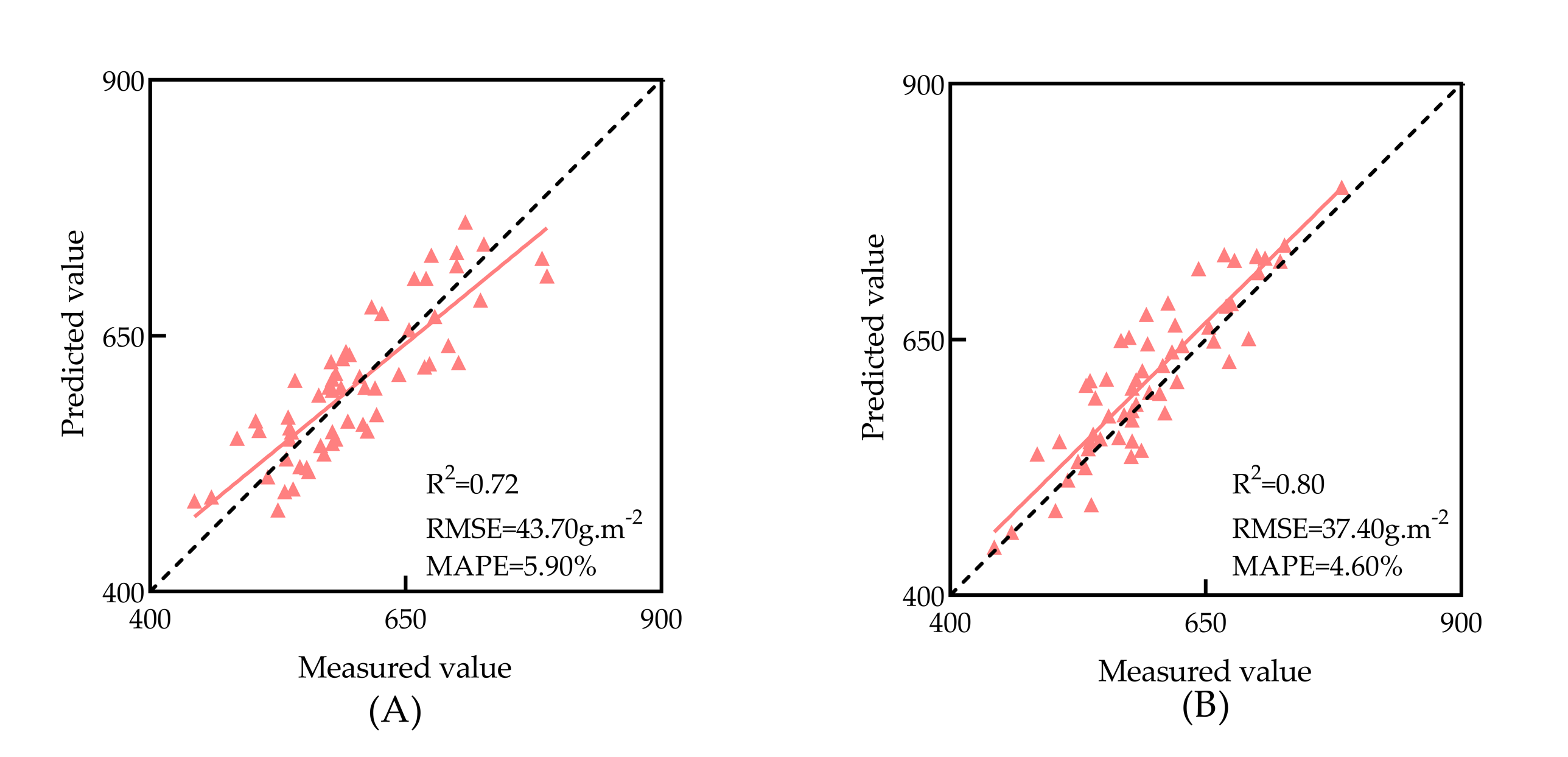

Supplement: Supplementary Figure 4 — Model test results based on the validation set 2: (A) MSR model, (B) RF model. [file Image_4.TIF]
